# Unilateral Parietal Bone Thickening in a Young Female Donor With a History of a Malignant Brain Neoplasm

**DOI:** 10.7759/cureus.98657

**Published:** 2025-12-07

**Authors:** Zoë M Rushetsky, Grace E Lynch, Victoria Pfennig, Caleb M Findley, Lynn M Waters

**Affiliations:** 1 Department of Osteopathic Medicine, Philadelphia College of Osteopathic Medicine, Suwanee, USA

**Keywords:** calvaria, hyperostosis, intracranial tumors, malignant brain neoplasm, parietal bone

## Abstract

Calvarial thickening can occur as a reaction to various intracranial conditions, most commonly due to chronic pressure from adjacent mass lesions. We report the case of a 21-year-old female cadaver found to have unilateral thickening of the right parietal bone during routine anatomy lab dissection. The available medical history noted a malignant brain tumor. Measurements showed the right parietal bone to be 7.2 mm thick, which is greater than what is considered normal. No other skeletal abnormalities were noted. The isolated thickening is most consistent with tumor-related osteoblastic activity. This case underscores the value of examining post-mortem bony changes to better understand intracranial disease.

## Introduction

The human skull is composed of the facial skeleton (viscerocranium) and the cranium (neurocranium), with the calvaria formed by the frontal, parietal, temporal, and occipital bones [1]. Focal calvarial thickening is an uncommon but clinically meaningful finding because it may signal underlying intracranial pathology, metabolic disease, trauma, or benign proliferative conditions. The prevalence of diffuse calvarial thickening in the general population is not well established and is typically found incidentally or post-mortem. 

Existing data largely comes from studies evaluating calvarial changes in the context of other conditions and the population studied. For example, in a case-control study assessing imaging features in patients without spontaneous intracranial hypotension, diffuse calvarial hyperostosis was present in approximately 13.2% of age- and sex-matched controls [2]. Although this example does not represent the general population, it highlights that mild or diffuse skull thickening can occur in otherwise asymptomatic individuals.

Isolated unilateral parietal thickening is considerably rarer and typically prompts evaluation for structural or pressure-related intracranial processes. A broad differential diagnosis exists for localized calvarial expansion. Intracranial mass lesions, particularly meningiomas, which produce hyperostosis in up to 20% of cases, are among the most recognized causes [3]. Additional considerations include hyperostosis frontalis interna and hyperostosis frontoparietalis [4,5], which are usually benign and most often observed in older adults, as well as metabolic or genetic bone disorders [6-9]. Intracranial calcified lesions such as “brain stones” may present incidentally but can mimic focal thickening on imaging and should be distinguished from true osseous remodeling [10,11]. Traumatic etiologies, including post-traumatic intradiploic arachnoid cysts, have also been shown to remodel the diploic space and alter the contour of the overlying calvaria over time [12,13].

In the setting of intracranial tumors, reactive calvarial thickening is believed to arise from chronic mechanical pressure, tumor-induced vascular changes, and stimulation of osteoblastic activity. Importantly, the extent of bony involvement often reflects the lesion’s duration and growth behavior rather than its histologic grade [6].

We report a young female cadaver with isolated unilateral thickening of the right parietal bone discovered during routine anatomical dissection. The documented history of a malignant intracranial neoplasm raises important considerations regarding the mechanisms and differential diagnosis of focal calvarial remodeling. The unilateral, localized manifestation in a young individual, with limited available clinical history, provides a valuable opportunity to contextualize how intracranial disease processes can imprint on post-mortem skeletal anatomy.

## Case presentation

A 21-year-old female cadaver was found to have unilateral thickening of the right parietal bone. This abnormality was observed during routine dissection performed in a gross anatomy laboratory at Philadelphia College of Osteopathic Medicine. The medical history of the donor was notable for having had an unspecified malignant brain tumor. The brain was liquified upon dissection, and therefore the neoplasm could not be visualized. The donor’s family and body donation program granted permission for the case to be published. 

**Figure 1:**
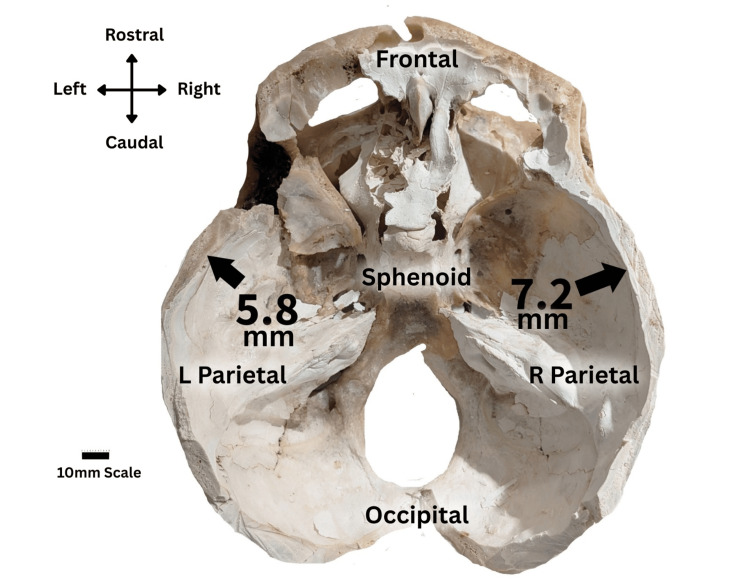
Horizontal calvarial section following removal of intracranial contents, showing right parietal bone thickening compared with the left side. A 10-mm scale bar is provided for measurement reference. The section exhibits a thickened parietal bone (7.2mm) on the right side and a normal parietal bone (5.8mm) on the left side.

Calvarial thickness was measured using a digital caliper. The right parietal bone measured 7.2 mm, compared with 5.8 mm on the left side (Figure 1). Although statistical significance cannot be established in a single cadaver, the degree of asymmetry exceeds the small side-to-side variation typically reported in adult parietal bone thickness. The measurements are presented descriptively to document the unilateral thickening observed in this case.

## Discussion

The typical parietal bone thickness in adults ranges from 2.85 to 6.93 mm [10]. The right parietal bone in this case measured 7.2 mm, exceeding the established upper limit of normal. Although the numerical difference from the left side (5.8 mm) is modest, the unilateral thickening above normative values suggests a focal pathologic process rather than normal variation.

Enlargement of the skull consists of various etiologies. One of many possibilities may be intradiploic arachnoid cysts. In rare instances, following physical trauma, cysts have the potential to form from herniation of the arachnoid space [13]. If erosion of the dura and diploic space has occurred, expansion of the cyst may lead to perceived focal thickening of the skull. Similar findings may occur in individuals with congenital abnormalities in the arachnoid membrane. Expansion of the CSF-filled space in coexistence with dural and diploic erosion of the scalp may lead to possible protrusions of the defect with overlaying skin and pericranium [13]. Given the lack of any diploic expansion, bony scalloping, or cystic cavities on inspection, this etiology is unlikely in our donor.

Bone continually remodels itself through a careful balance between the activity of osteoblasts, which build it up, and osteoclasts, which break it down. Regional disturbance of the balance, caused by a tumor pressing on the bone, can result in increased local bone spiculae [11]. The thickening of the right parietal bone in this case implies that the lesion was likely located ipsilateral to the thickened parietal bone.

Meningiomas are the most common primary benign intracranial tumors, representing about 37.6% of all brain tumors [14]. They originate from the arachnoid mater and may grow in size outside of the dura. They can cause thickening (hyperostosis) of the cranial bones [6]. Calvarial thickening is present in up to two-thirds of intraosseous meningiomas [6]. Known risk factors for these brain tumors include oral contraceptive use, neurofibromatosis type II, and breast cancer [7]. Although classic meningiomas are benign, atypical and anaplastic variants (WHO grades II-III) behave more aggressively with increased invasion, recurrence, and potential for destructive osseous changes rather than the smooth hyperostosis seen in grade I benign tumors [3,14]. In this case, however, the donor’s documented history of a malignant intracranial neoplasm makes a typical benign meningioma less likely, though rare malignant or atypical meningioma variants cannot be excluded without any histopathology.

Tuberous sclerosis complex (TSC) is another syndrome that causes structural cranial changes, with the thickened frontal and temporal bones being among those observed. TSC is a neurocutaneous syndrome defined by hamartomas in multiple organs, including the brain, kidneys, heart, and skin [15]. A recent study demonstrated that TSC patients had significantly thicker skulls than controls, which were associated with subcortical calcifications. Notably, thickened skull in TSC does not appear to be associated with age, sex, or anticonvulsant therapy [16]. In this case, the absence of hamartomas or calcification makes TSC less probable.

Skeletal dysplasias, a group of more than 450 different genetic disorders that can affect the development or growth of bone and cartilage, may also result in anomalous cranial morphology [17]. Achondroplasia, the most frequent form of this condition, is characterized by calvarial thickening, craniofacial abnormalities, and wormian bones (abnormally shaped small bones found between the cranial sutures) [9]. These malformations can be associated with posterior fossa cranial nerve compression or flow perturbation of the cerebrospinal fluid [18]. In this case, the skull was devoid of wormian bones or any other structural modifications that would be typical for skeletal dysplasia.

Hyperostosis frontalis interna (HFI) is described as an abnormal bone growth of the posterior portion of the inner table of the frontal bone. If the thickening extends into the parietal bones, it is termed hyperostosis frontoparietalis. The etiology of HFI is suggested to stem from sex hormone dysregulation and aging, with the majority of cases seen in elderly, postmenopausal, and nulliparous women [4,5]. Although knowledge of our donor’s medical history is limited, this was a 21-year-old nulliparous female with calvarial thickening observed in the parietal bone only, making HFI less likely. 

Intracranial calcifications, often referred to as “brain stones”, can also influence the surrounding calvaria. These densely calcified lesions typically form slowly in response to longstanding inflammation, vascular injury, or other chronic intracranial disturbances. Furthermore, as they expand or persist over time, they may create subtle, repetitive irritation along the inner skull. Chronic stimulation can trigger a reactive osteoblastic response, leading to gradual thickening of the overlying bone known as hyperostosis [11]. While no intracranial calcifications were documented in this donor, the absence of full imaging prevents complete exclusion.

Lastly, acromegaly is an endocrine disorder caused by increased growth hormone. It is commonly the result of benign pituitary adenomas and may also result in skull thickening. The increase in insulin-like growth factor 1 (IGF-1) levels enhanced bone cell formation and decreased normal cell turnover [8]. Typical osteological features include malformation of the skull, jaws, and sinuses [19]. Patients with acromegaly have been found to have significantly enlarged skull diameters in anterior-posterior, as well as in lateral dimensions [20]. As the other skeletal characteristics of acromegaly were not present in this donor, this is an unlikely diagnosis.

Overall, given the donor’s documented history of a malignant intracranial neoplasm, the asymmetry in parietal bone thickness raises the possibility, though not definitive proof, of tumor-related calvarial remodeling. The localized thickening above normative values, in combination with the known malignant intracranial process, therefore remains the most plausible explanation based on the information available.

## Conclusions

The current case describes a 21-year-old female donor with unilateral thickening of the right parietal bone and a documented history of an intracranial malignant neoplasm. Longstanding pressure from intracranial tumors is known to initiate extensive bone formation, presenting as hyperostosis of the calvaria. Although focal calvarial remodeling is well described in association with intracranial tumors, through mechanisms such as chronic pressure, altered vascular dynamics, and stimulation of osteoblastic activity, the precise cause of the thickening in this donor cannot be definitively established due to limited clinical and pathological information. Nevertheless, the presence of unilateral thickening above normative values, in conjunction with the recorded history of a brain tumor, makes tumor-related remodeling a plausible explanation.

This case highlights the importance of recognizing focal calvarial changes as a potential indicator of underlying intracranial or systemic disease. Even subtle or isolated bony abnormalities can contribute to differential diagnosis when integrated with clinical history and imaging. By documenting post-mortem variations such as this one, anatomy-based investigations can help expand the spectrum of known presentations and remind clinicians, radiologists, and anatomists to consider a broad range of etiologies, including neoplastic, metabolic, traumatic, and benign proliferative processes, when evaluating focal cranial thickening.
